# The Effect of L‐Carnitine Supplementation in Culture Medium on the Development and Cryopreservation of in Vitro Produced Bovine Embryos

**DOI:** 10.1002/vms3.70376

**Published:** 2025-04-24

**Authors:** Mehmet Yildiz, Yunus Cetin

**Affiliations:** ^1^ Department of Obstetrics and Gynecology Faculty of Veterinary Medicine Van Yuzuncu Yil University Van Turkey; ^2^ Department of Obstetrics and Gynecology Faculty of Veterinary Medicine Burdur Mehmet Akif Ersoy University Burdur Turkey

**Keywords:** bovine, embryo, IVP, L‐carnitine, slow freezing, vitrification

## Abstract

**Background:**

In vitro culture media play a crucial role in supporting the growth and development of bovine embryos. Despite advancements in media formulations, there is ongoing interest in optimising these media by supplementing them with compounds like L‐carnitine to improve embryo development and cryopreservation outcomes. However, the effects of L‐carnitine supplementation in commercial culture media remain unclear. The aim of this study was to investigate the effects of adding 0.75 mM L‐carnitine to commercial in vitro culture (IVC) media on bovine in vitro embryo production (IVP). The first phase of the study evaluated the effects of L‐carnitine on embryo development, while the second phase evaluated the effects of L‐carnitine on embryo cryopreservation.

**Methods:**

A total of 508 bovine ovaries used in the study were harvested from slaughterhouses. Only morphologically healthy ovaries were included in the study, while ovaries with cysts or any pathological findings were excluded. Oocytes were collected by aspiration method. The collected oocytes were fertilised in vitro using prepared spermatozoa under standard conditions. Following fertilisation, embryos were morphologically evaluated, and only those exhibiting uniform blastomeres and minimal cytoplasmic fragmentation were included in subsequent steps. The embryos were then randomly divided into two groups: one supplemented with 0.75 mM L‐carnitine in the IVC medium and the other without supplementation. Cleavage rates on Day 4 and embryo development rates on 7 days after fertilisation were evaluated with the supplementation of L‐carnitine. In addition, the survival effects of embryos collected on Day 7 after direct culture, slow freezing and vitrification were investigated.

**Results:**

It was determined that L‐carnitine supplementation to IVC medium did not affect the cleavage rates on Day 4 and blastocyst development rates on Day 7 (*p* > 0.05). Moreover, it did not affect the survival and development rates of embryos collected on Day 7 following both slow freezing and thawing, as well as vitrification and warming processes (*p* > 0.05).

**Conclusions:**

Supplementation of L‐carnitine to commercially available in vitro culture medium did not enhance embryo development rates or survival rates after cryopreservation. This is likely due to the presence of antioxidant compounds in commercial embryo culture media.

## Graphical abstract

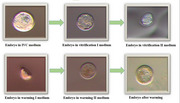

## 1 Introduction

Embryo transfer technologies have advanced significantly in recent years, enabling the large‐scale production of bovine embryos. According to the 2022 data from the International Embryo Transfer Society, approximately 2,011,480 bovine embryos are produced annually worldwide, with 80.39% produced in vitro and 19.61% derived in vivo. While 35.2% of embryos collected of in vivo derived (IVD) are transferred fresh, 55.7% of embryos in vitro produced (IVP) are transferred fresh. The fresh transfer of IVP embryos may be due to their susceptibility to cryopreservation (Ferré et al. 2020; Viana 2023). This sensitivity to cryopreservation may result from abnormal oocytes, high cytoplasmic lipid content, different lipid droplet arrangements and low inner cell mass (del Collado et al. 2016; Seidel 2006).

Modifying in vitro culture (IVC) conditions, such as eliminating serum supplements, has been shown to reduce lipid accumulation and improve cryotolerance in IVP embryos. However, this approach may compromise blastocyst development (Accorsi et al. 2016; Figueiró et al. 2020). To address these challenges, L‐carnitine, a quaternary ammonium compound that facilitates the transport of fatty acids into mitochondria for β‐oxidation (Somfai et al. 2011), has gained attention. L‐carnitine supplementation during IVC has been reported to decrease lipid accumulation in embryos without the use of lipolytic agents (Murillo et al. 2017). Moreover, it has been demonstrated to enhance the survival and in vitro development of bovine IVP embryos following cryopreservation (Takahashi et al. 2013; Held‐Hoelker et al. 2017; Ruiz et al. 2014).

Another critical factor influencing embryo quality is the level of reactive oxygen species (ROS). ROS levels may increase in oocytes cultured at atmospheric oxygen (20% O_2_) level (Ali et al. 2003) or during embryo cryopreservation (Ahn et al. 2002). Excessive ROS production is reported to impair ß‐oxidation, affecting mitochondrial function and leading to lipid accumulation (Van Hoeck et al. 2015). Elevated intracellular ROS levels result in various cellular damages, including apoptosis and DNA fragmentation, which in turn lead to embryo fragmentation and developmental blocks (Guerin et al. 2001). Therefore, the addition of antioxidants such as L‐carnitine to the IVC medium supports mitochondrial function, reduces ROS levels and protects DNA from fragmentation. Thus, it positively affects embryo development and protects embryos against cryodamage by reducing ROS levels during cryopreservation (Silva et al. 2015; Somfai et al. 2011).

Despite evidence supporting the beneficial effects of L‐carnitine on embryo survival and development (Takahashi et al. 2013; Held‐Hoelker et al. 2017; Ruiz et al. 2014), the optimal timing, concentration of L‐carnitine added to the IVC medium, and the number of cryoprotectants used during cryopreservation have not yet been standardised. Commercial embryo culture media are currently employed on a widespread basis in the production of embryos in vitro. However, there is a lack of research evaluating the effects of L‐carnitine supplementation in commercial bovine embryo culture media. Embryo production and cryopreservation involve multiple stages, including in vitro maturation (IVM), in vitro fertilization (IVF), IVC, cryopreservation and thawing or warming. Each stage's media composition, particularly the concentrations of supplements and cryoprotectants, can significantly influence embryonic development and survival. The aim of this study was to investigate the effects of adding 0.75 mM L‐carnitine to a commercial IVC medium. In this context, (1) division rates of embryos at Day 4, (2) development of embryos at Day 7 after fertilisation, (3) development of embryos after Day 7, (4) survival and development of slow frozen embryos after thawing and (5) survival and development of vitrified and heated embryos were investigated.

## 2 Materials and Methods

The IVM, IVF and IVC media used in this study (IVF Bioscience, Falmouth, UK) and all other chemicals and reagents, unless otherwise specified, were obtained from Sigma‐Aldrich (St. Louis, MO, USA). The study was conducted between October 2020 and August 2021.

Ovaries used in the study were obtained from Holstein and Simmental cattle slaughtered at two different abattoirs in Burdur, Turkey. Ovaries were collected randomly without consideration of the pedigree or general characteristics of the cows. However, ovaries showing cysts or pathological conditions were excluded from the study. All procedures, including in vitro embryo production and cryopreservation, were carried out at the Genetics and Embryo Technologies Application and Research Center of Burdur Mehmet Akif Ersoy University.

### 2.1 Oocyte Collection, Maturation and Fertilization

Bovine ovaries derived from the slaughterhouse were brought to the laboratory within 2 h at the latest. For aspiration medium, a tissue culture medium (TCM199) containing 1% foetal bovine serum (FBS) was used. Oocytes were aspirated from follicles measuring approximately 2–8 mm in diameter using 10 mL syringes equipped with sterile 18‐gauge needles. Only Grade A and B immature oocytes were used for the maturation process (Kakkassery et al. 2010). A total of 40–45 oocytes were matured in 500 µL IVM medium in Petri dishes with wells in the centre. The maturation process was carried out by incubating for 20–22 h. The evaluation was conducted according to Kobayashi et al. (1994). In the study, to avoid variations due to different bulls, frozen sperm from a single bull was used for fertilisation. Matured oocytes were fertilised using the conventional IVF method. The fertilisation was carried out by placing 2 × 10^6^/mL spermatozoa and 40–45 oocytes in 500 µL IVF medium in petri wells and incubating for 24 h. Throughout all stages of IVM, IVF and IVC media were covered with mineral oil to prevent evaporation and contamination. In addition, all incubation processes were carried out at 39°C in an atmosphere of 5% CO_2_, 5% O_2_, 90% N_2_ and high humidity.

### 2.2 Effects of L‐Carnitine Cleavage and Embryo Development

For Experiments 1 and 2, presumptive zygotes were randomly divided into two groups to be cultured in either L‐carnitine‐containing (Group IVC‐LC) or L‐carnitine‐free (Group IVC) culture medium. L‐carnitine was added to the Group IVC‐LC at a concentration of 0.75 mM (Zolini et al. 2019). The medium was prepared by placing 40–45 embryos per 500 µL in a petri dish with a centre well. Presumptive zygotes were cultured in the incubator until Day 4 post‐fertilisation, and cleavage rates were evaluated. Unfertilised or non‐cleaved oocytes were excluded from the study. Embryo development rates were evaluated on Day 7 post‐fertilisation. The experiment was repeated 22 times.

### 2.3 Effects of L‐Carnitine on the Development of Blastocysts

For Experiment 3, on the 7th day after fertilisation, early blastocysts and blastocysts were cultured for 96 h in Group IVC‐LC and Group IVC. The rates of embryo expansion, hatching and hatched blastocysts were evaluated at 24, 48 and 72 h. The experiment was repeated 11 times.

### 2.4 Effects of L‐Carnitine on the Development of Slow‐Frozen and Thawed Blastocysts

For Experiment 4, early blastocysts and blastocysts cultured in Group IVC‐LC and Group IVC media on Day 7 post‐fertilisation were harvested and subjected to slow freezing at a controlled rate. The slow‐freezing medium consisted of TCM‐199 containing 1.5 M Ethylene Glycol (EG), 0.1 M sucrose and 20% FBS. Harvested embryos were transferred to 0.25 mL straws, 1 embryo per straw. After equilibrating the embryos in the slow‐freezing solution at room temperature for 10 min, they were placed in an adjustable‐rate freezing device (Freeze Control CL‐5500). The protocol involved equilibrating at −6°C for 10 min. Two minutes after placing the straws in the device, seeding was performed using a cooled cotton swab. The freezing process was carried out at a rate of −0.3°C to −6°C per minute until reaching −32°C. Upon completion, the straws were transferred to goblets and stored in a liquid nitrogen tank for at least one day.

The slow‐frozen embryos were thawed by holding the straws in the air for 5 s, followed by immersion in a 30°C water bath for 20 s. Thawed embryos were washed three times in TCM‐199 medium containing 0.3 M sucrose and 10% FBS, followed by washing in TCM‐199 medium with 10% FBS. Subsequently, embryos were cultured in Group IVC‐LC and Group IVC media for 96 h. The rates of expanded, hatching and hatched blastocysts of thawed embryos were evaluated at 24, 48 and 72 h. The experiment was repeated six times.

### 2.5 Effects of L‐Carnitine on the Development of Blastocysts Post‐Vitrification

For Experiment 5, early blastocysts and blastocysts cultured in Group IVC‐LC and Group IVC media on Day 7 post‐fertilisation were vitrified. The vitrification medium was prepared in two stages. Firstly, embryos were washed three times in the first vitrification medium containing 3.5 M EG and 20% FBS in TCM‐199. The samples were then washed three times in the second vitrification medium, which contained 7 M EG, 0.5 M sucrose, 18% (w/v) Ficoll 70 and 20% FBS in TCM‐199, before vitrification. Harvested embryos were transferred to 0.25 mL pipettes with a 10 µL drop of second vitrification solution, 1 embryo per pipette. The open end of the straw was sealed with a plug and vitrified by immersion in liquid nitrogen within 45 s. After vitrification, the straws were placed in goblets and stored in a liquid nitrogen tank for at least one day.

Vitrified embryos were warmed in the same way as the thawing procedure for slow‐frozen embryos. The embryos were first washed in TCM‐199 medium containing 0.5 M sucrose and 20% FBS, then in TCM‐199 medium with 0.25 M sucrose and 20% FBS and finally in TCM‐199 medium with 20% FBS, with each wash performed at least three times. The embryos were then cultured in their respective media, Group IVC‐LC and Group IVC, for 96 h. The rates of expanded, hatching and hatched blastocysts were evaluated at 24, 48 and 72 h. The experiment was repeated five times.

### 2.6 Statistical Analysis

The normality of the numerical variables was checked using the Shapiro–Wilk test. For Experiments 1–5, the independent samples *t*‐test was used for groups with normal distributions. For groups where normality was not achieved, the Mann–Whitney *U* test was applied. Statistical significance was considered at a level of *p* < 0.05 for all calculations and interpretations, with hypotheses being two‐tailed. The statistical analysis of the data was performed using the SPSS v26 (IBM Inc. Chicago, IL, USA) statistical package.

## 3 Results

### 3.1 Oocyte Collection, Maturation and Fertilization

From 508 ovaries collected under appropriate conditions at slaughterhouses, a total of 1258 oocytes were harvested. Among these, 29.89% were classified as first‐degree mature, and 65.02% as second‐degree mature, resulting in 1194 mature oocytes used for fertilisation. The remaining 64 oocytes (5.09%) did not reach maturity and were excluded from the fertilisation process. Only oocytes classified as first‐ and second‐degree mature were included in the subsequent procedures.

After 24 h post‐fertilisation, cells were vortexed for the denudation process. Due to loss and degeneration, 104 cells (8.71%) were excluded from the study.

### 3.2 Experiments 1–2: The Effect of L‐Carnitine on Cleavage and Embryo Development

Of the 1090 mature oocytes on Day 4 after IVF, unfertilised oocytes (UFO) and 2‐cell embryos were excluded from the study. The study was continued with a total of 465 embryos with 4, 8 and more cells (Table 1). On the 7th day of IVF, there were 233 embryos in Group IVC and 232 embryos in Group IVC‐LC. The development rates of the embryos are shown in Table 2.

**TABLE 1:** Cleavage rates on Day 4 of in vitro fertilization.

Group	Total number of mature oocytes (n)	UFO %	2 cell embryo (%)	4 cell embryo (%)	>8 and more cell embryo (%)	Total embryo (%)
**Group IVC**	553	38.4±2.8	15.9 ± 1.5	25.8 ± 2.5	19.9 ± 3.0	60.6 ± 2.7
**Group IVC‐LC**	537	39.3± 2.9	14.6± 2.2	25.2 ± 2.9	20.9 ± 2.4	60.7 ± 2.9
***p***		0.906	0.557	0.655	0.581	0.888

*Note*: Percentage results are presented as means ± SEM.

**TABLE 2:** Findings on Day 7 of in vitro fertilization.

	Number of total embryos d 4 (n)	Degenerated embryos (%)	Morula (%)	Early blastocyst (%)	Blastocyst (%)	Advanced blastocyst (%)	Total embryo d 7 (%)
**Group IVC**	233	28.7±3.8	16.6 ±3.4	13.3 ±3.3	37.2 ±3.8	4.2 ±1.7	71.3 ±3.8
**Group IVC‐LC**	232	32.3 ± 4.2	13.0 ± 3.3	9.9± 3.1	43.1± 4.1	1.8± 1.0	67.7± 4.2
***p***		0.638	0.420	0.322	0.359	0.244	0.638

*Note*: Percentage results are presented as means ± SEM.

### 3.3 Experiment 3: Effect of L‐Carnitine on the Embryo Development

On Day 7 post‐fertilisation, embryos that had reached the early blastocyst and blastocyst stages were cultured in the same medium and evaluated at 24, 48 and 72 h. Supplementation of L‐carnitine to the IVC medium did not enhance the expanded blastocyst rates (*p* > 0.05). Although a significant decrease was observed in hatching/hatched blastocyst ratios at 48 h (*p* < 0.05), this difference disappeared at 72 h (*p* > 0.05). Thus, L‐carnitine did not affect the development rates of embryos cultured without freezing (Table 3).

**TABLE 3:** Developmental status of early blastocysts and blastocysts cultured directly.

		Expanded blastocyst (%)			Hatching/Hatched blastocyst (%)		
	Number of embryos	24 h	48 h	72 h	24 h	48 h	72 h
**Grup IVC**	62	35.5 ± 9.9	43.0 ± 9.6	55.9 ± 11.0	16.7 ± 9.5	30.2 ± 10.3	31.7 ± 9.9
**Grup IVC‐LC**	57	31.7 ± 12.1	34.6 ± 12.2	55.2 ± 14.1	0.5 ± 0.5	4.2 ± 3.6	10.9 ± 5.8
***p***		0.589	0.506	0.946	0.098	0.020^*^	0.058

* *p* < 0.05.

### 3.4 Experiment 4: Effects of L‐Carnitine Development of Slow‐Frozen and Thawed Blastocysts

On Day 7 post‐fertilisation, early blastocysts and blastocysts were slow‐frozen and then thawed before being cultured back again in the same IVC medium. During thawing, it was observed that one embryo in the Group IVC and one embryo in the Group IVC‐LC were fragmented. The developmental progress of the embryos was evaluated at 24, 48, and 72 h post‐thawing (Table 4).

**TABLE 4:** Comparison of development rates of slow‐frozen and thawed embryos.

	Number of embryos	Expanded blastocyst (%)			Hatching/Hatched blastocyst (%)		
		24 h	48 h	72 h	24 h	48 h	72 h
**Grup IVC**	33	9.3±6.0	13.0 ±8.3	19.0 ±9.4	0.0 ±0.0	5.6 ±5.6	5.6±5.6
**Grup IVC‐LC**	36	22.2± 8.0	49.3± 16.9	49.3± 16.9	0.0± 0.0	12.5± 8.5	20.8± 10.0
***p***		0.198	0.113	0.164	—	0.528	0.214

### 3.5 Experiment 5: Effects of L‐Carnitine on the Development of Blastocysts Post‐Vitrification

On the 7th day post‐fertilisation, early blastocysts and blastocysts were vitrified and then thawed before being re‐cultured in the same IVC medium. During the thawing process, it was observed that one embryo in the Group IVC and two embryos in the Group IVC‐LC were fragmented. The developmental progress of the embryos was evaluated at 24, 48 and 72 h post‐warming (Table 5).

**TABLE 5:** Comparison of development rates of vitrified and thawed embryos.

	Number of embryos	Expanded blastocyst (%)			Hatching/Hatched blastocyst (%)		
		24 h	48 h	72 h	24 h	48 h	72 h
**Grup IVC**	26	10.5 ± 7.7	10.5±7.7	14.5 ±11.6	6.5 ±4.2	10.5 ±7.7	10.5±7.7
**Grup IVC‐LC**	31	34.2±9.0	34.2 ±9.0	36.7± 7.8	10.9± 7.8	19.6± 6.9	23.6± 4.9
***p***		0.056	0.056	0.090	0.814	0.278	0.110

## 4 Discussion

L‐carnitine has been widely studied for its potential to enhance oocyte quality, embryo development and cryopreservation outcomes in various mammalian species. Previous studies have shown that L‐carnitine can improve lipid metabolism, reduce cytoplasmic lipid accumulation and enhance cryotolerance in bovine embryos (Held‐Hoelker et al. 2017; Sudano et al. 2014). In this study, the effect of L‐carnitine addition (0.75 mM) to the commercial IVC medium on the cryopreservation of IVP embryos was investigated.

Carrillo‐González et al. (2023) reported that the supplementation of 1.5 mM L‐carnitine to the IVC did not significantly affect cleavage rates of bovine embryos cultured at 38.5°C and 5% CO_2_, with rates of 68.3% in the control group and 67.1% in the L‐carnitine group. Similarly, Zolini et al. (2019) found no increase in cleavage rates when 0.75 mM L‐carnitine was added to IVC, with cleavage rates of 76.4% and 78.8% in the L‐carnitine and control groups, respectively. Consistent with these studies, our results showed no significant difference in cleavage rates: 60.7% in the L‐carnitine group and 60.6% in the control group. These differences across studies may be attributed to factors such as L‐carnitine concentration, IVC media composition, methods of oocyte retrieval, the quality of selected oocytes and laboratory conditions.

Zolini et al. (2019) also observed no effect of L‐carnitine (0.75 mM) on blastocyst development by Day 7, with control and L‐carnitine groups exhibiting rates of 22.2% and 20.6%, respectively. However, they noted that increasing the L‐carnitine concentration to 3.03 mM resulted in a decrease in blastocyst development. In contrast, Takahashi et al. (2013) found that IVC supplementation with 1.518 mM and 3.03 mM L‐carnitine enhanced blastocyst development rates to 40.2% and 44.6%, respectively, compared to 32.3% in the control group. Our study found blastocyst development rates of 43.1% in the L‐carnitine group and 36.9% in the control group, with the former being higher, though no statistically significant difference was observed. This discrepancy might be due to the exclusion of non‐dividing and 2‐cell embryos on Day 3 in our study, ensuring only embryos with at least four cells were used for further development.

Theoretically, antioxidants have positive effects on bovine embryo development. However, some researchers (Fujitani et al. 1997; Iwata et al. 1998) have found that embryo development is adversely affected depending on oxygen levels or culture conditions. Apart from the studies by De Matos et al. (2000) and Olson and Seidel (2000), the positive effects of antioxidants are primarily observed under 20% oxygen conditions. IVC also plays a crucial role in achieving positive results. For example, 50–100 µM cysteamine is used in IVC with TCM199 medium (De Matos et al. 1996), while 25–50 µM cysteamine is sufficient in SOF medium (De Matos et al. 2000). Presumably, this difference may be due to the presence in the SOF medium of factors that protect against free radical damage. (Morales et al. 1999). In our study, we found that after Day 7 of IVF, L‐carnitine did not affect the expanded blastocyst rates at 24, 48 and 72 h. Interestingly, hatching blastocyst rates in the L‐carnitine group were statistically lower than those in the control group at 48 h, although no significant differences were observed at 72 h. The commercial IVC medium used in the present study is reported to be supplemented with BSA, vitamins, amino acids and antioxidants. However, the concentrations of the substances used were not disclosed. Therefore, it is speculated that adding additional antioxidants to a medium already containing antioxidants could increase the antioxidant concentration and adversely affect embryonic development. The lower hatching rates at 48 h suggest that this may be due to the 5% O_2_ incubator conditions or the concentration of L‐carnitine used. Future studies using defined media rather than commercial media, with varying concentrations of L‐carnitine and different O_2_ levels, could yield new insights. In addition, since ROS levels were not measured in this study, the lack of difference between the control group and the L‐carnitine group could be due to similar ROS values. Evaluating ROS levels and apoptosis rates in future studies could contribute to a better understanding of L‐carnitine's efficacy.

Supplementation with L‐carnitine in IVC has been reported to enhance certain aspects of embryo cryotolerance. Zolini et al. (2019) demonstrated that 0.75 mM L‐carnitine increased re‐expansion rates in slow‐frozen embryos but had no significant effect on hatching blastocyst rates. Similarly, Takahashi et al. (2013) found that higher concentrations of L‐carnitine (1.5 mM and 3.03 mM) improved the survival rates of slow‐frozen embryos. In our study, L‐carnitine supplementation to the IVC did not statistically affect the rates of expanded and hatching/hatched blastocysts post‐thawing of slow‐frozen embryos. However, there was a proportional increase in embryo development at 24, 48 and 72 h post‐thawing in the L‐carnitine supplemented group. This proportional increase suggests that significant differences might emerge with larger sample sizes, underscoring the importance of further research. Due to its role in lipid metabolism, L‐carnitine supplementation in IVC can enhance the cryotolerance of embryos (Held‐Hoelker et al. 2017; Takahashi et al. 2013). It is reported that supplementing the embryo IVC, supplementation with lipid‐free BSA, with L‐carnitine enhances survival post‐thawing without affecting embryo lipid content (Held‐Hoelker et al. 2017). The lack of difference in the rates of expanded, hatching and hatched blastocysts post‐thawing in our study is consistent with Held‐Hoelker et al. (2017). It is reported that adding L‐carnitine to the IVC of IVP embryos obtained from cattle reduces lipid content (Takahashi et al. 2013), which is associated with the cryo‐tolerance of IVP embryos. Our results suggest that the absence of a detectable impact may be due to comparable lipid profiles across groups. ROS levels increase during freezing and thawing (Ahn et al. 2002) and the increased ROS can cause damage or death to embryos (Valencia and Moran 2004). The numerical increase in embryonic development in the L‐carnitine IVC group in our study supports the findings of Ahn et al. (2002), suggesting that L‐carnitine supplementation in IVC might reduce ROS levels. Therefore, repeating the studies with larger sample sizes and measuring ROS levels in embryos could yield more meaningful results. The creation of pregnancies after the transfer of frozen‐stored embryos is more complex than the survival measurements observed in IVC (Zolini et al. 2019). Therefore, we believe that new approaches to improve embryo cryotolerance need to include not only in vitro evaluation but also transfers to assess pregnancy rates.

Gupta et al. (2010) reported that vitrification increases ROS levels, which reduces the survival and developmental competence of pig oocytes or embryos. Adding acetyl‐L‐carnitine to the maturation and vitrification medium enhances the membrane of mitochondrial potential and modifies the lipid profile of the membrane in buffalo oocytes, thereby improving embryo enhancement and cryotolerance (Xu et al. 2019). In particular, L‐carnitine supplementation during bovine oocyte maturation distributes lipid content from the centre to the periphery (Chankitisakul et al. 2013). An increase in the amount of cytoplasmic lipid droplets in oocytes makes them more susceptible to cold damage in the course of cryopreservation. The mechanical removal of lipid droplets or their reduction with chemical agents can improve the cryotolerance of oocytes and embryos (Ushijima et al. 1996). Zolini et al. (2019) found that adding 0.75 mM L‐carnitine to the IVC enhanced cryotolerance after warming, but did not affect implantation and pregnancy rates following the transfer of frozen‐stored bovine embryos. In our study, it was found that adding L‐carnitine to the IVC did not statistically enhance the rates of expanded blastocysts, hatching and hatched blastocysts after warming in vitrified embryos. However, there was a notable proportional increase in embryo development at 24, 48 and 72 h in the L‐carnitine‐supplemented group. The lipid content of the embryos used in our study was not analysed, so the absence of a difference might be due to the similar lipid content of the embryos, supporting Carrillo‐González et al. (2023). Increased ROS amount can cause damage or death in oocytes and embryos (Valencia and Moran 2004). The numerical increase in embryonic development in the L‐carnitine group in our study might be due to L‐carnitine's effect in reducing ROS levels. Therefore, more meaningful results might be obtained in future studies by increasing the number of materials and measuring ROS levels in embryos.

## 5 Conclusion

Supplementation of L‐carnitine to commercially available IVC did not improve embryo development or survival rates after cryopreservation in the present study. The presence of antioxidants in the commercial medium may have masked the potential effects of L‐carnitine supplementation, as antioxidants are known to influence oxidative stress levels in embryos (De Matos et al. 2000; Olson and Seidel 2000). Future studies should include the measurement and evaluation of lipid content and ROS levels in embryos cultured in commercial media. Further future studies could aim to investigate optimal L‐carnitine doses in commercial media to enhance embryo survival and development. Research should focus on identifying the most effective concentrations of L‐carnitine that could improve cryotolerance, taking into account the influence of other medium components, such as antioxidants, which might interact with L‐carnitine supplementation. Although significant results have been achieved in laboratory settings, it is recommended that future studies further evaluate the efficacy of L‐carnitine supplementation by assessing pregnancy rates after embryo transfer following cryopreservation and thawing. This would provide a more comprehensive understanding of L‐carnitine's impact on embryo viability and development under in vivo conditions, ultimately improving its application in assisted reproductive technologies.

## Author Contributions

**Mehmet Yildiz**: conceptualisation, data curation, investigation, methodology, software, validation, visualisation, writing – original draft, writing – review and editing. **Yunus Cetin**: conceptualisation, methodology, funding acquisition, investigation, project administration, resources, writing – original draft, writing – review and editing, supervision.

## Ethics Statement

All procedures conducted in this experiment received approval from the Animal Experiments Local Ethics Committee at Burdur Mehmet Akif Ersoy University Rectorate (Ethical confirmation number: 626).

## Conflicts of Interest

The authors declare no conflicts of interest.

## Data Availability

The data that support the findings of this study are available from the corresponding author upon reasonable request.
